# BDNF-loaded chitosan-based mimetic mussel polymer conduits for repair of peripheral nerve injury

**DOI:** 10.3389/fcell.2024.1431558

**Published:** 2024-07-01

**Authors:** Lei Li, Ziyue Chu, Shihao Li, Tong Zheng, Shusheng Wei, Yunpeng Zhao, Peilai Liu, Qunshan Lu

**Affiliations:** ^1^ Department of Adult Joint Reconstructive Surgery, Beijing Jishuitan Hospital, Capital Medical University, Beijing, China; ^2^ Department of Orthopedics, Qilu Hospital of Shandong University, Jinan, Shandong, China

**Keywords:** mimetic mussel, polymer conduits, BDNF, peripheral nerve injury, chitosan-based

## Abstract

Care for patients with peripheral nerve injury is multifaceted, as traditional methods are not devoid of limitations. Although the utilization of neural conduits shows promise as a therapeutic modality for peripheral nerve injury, its efficacy as a standalone intervention is limited. Hence, there is a pressing need to investigate a composite multifunctional neural conduit as an alternative treatment for peripheral nerve injury. In this study, a BDNF-loaded chitosan-based mimetic mussel polymer conduit was prepared. Its unique adhesion characteristics allow it to be suture-free, improve the microenvironment of the injury site, and have good antibacterial properties. Researchers utilized a rat sciatic nerve injury model to evaluate the progression of nerve regeneration at the 12-week postoperative stage. The findings of this study indicate that the chitosan-based mimetic mussel polymer conduit loaded with BDNF had a substantial positive effect on myelination and axon outgrowth. The observed impact demonstrated a favorable outcome in terms of sciatic nerve regeneration and subsequent functional restoration in rats with a 15-mm gap. Hence, this approach is promising for nerve tissue regeneration during peripheral nerve injury.

## 1 Introduction

Peripheral nerve injury (PNI) primarily arises from mechanical challenges, such as natural disasters, wars, and motor vehicle crashes, which have long impaired the sensory function of patients, complicated everyday activities, and degraded their quality of life (Sullivan et al., 2016; Jiang et al., 2023). Therefore, there is a pressing need for expeditious and efficacious restoration of peripheral nerve damage in clinical practice. In the United States, billions of dollars are spent each year to treat and repair damaged peripheral nerve injuries (Schmidt and Leach, 2003; Liu et al., 2022). Autografting is widely regarded as the prevailing technique for peripheral nerve repair and is the benchmark in clinical nerve regeneration (Lundborg, 2000; Grinsell and Keating, 2014). The material originates from bodily tissue, which plays a pivotal role in directing the regeneration of nerve fibers, promoting the survival of Schwann cells, and facilitating the spread of neurotrophic factors to the site of injury (Carvalho, Oliveira, and Reis, 2019). However, this method has some limitations that limit its application, including limited donor materials and loss of function, multiple surgical operations, mismatch between graft and nerve tissue size, and postoperative complications such as scar tissue invasion due to the migration of fibroblasts into the separation zone. Therefore, it is difficult to repair defective peripheral nerves (Scheib and Hoke, 2013; Liu et al., 2019; Manoukian et al., 2020; Smith et al., 2022). Although the use of allografts can partially solve these problems, other systemic side effects, such as immune rejection and secondary infection, still exist (Huang et al., 2012; Dixon et al., 2018).

Tissue engineering of neural conduits offers a viable solution for facilitating the restoration of nerve abnormalities characterized by substantial gaps (Lee and Wolfe, 2012; Meyer et al., 2016; Peng et al., 2017; Lu et al., 2021). The fundamental biological properties of nerve conduits include biocompatibility, suitable mechanical attributes, and the ability to facilitate neuronal behavior to direct the growth trajectory of nerve cells (Tatard et al., 2005; Zhu et al., 2018; Parker et al., 2021). These conduits work as a means to connect areas that have been destroyed, improve the effectiveness of nerve regeneration, and successfully restore motor and sensory functioning in the affected region. This is achieved through the promotion of angiogenesis, cell proliferation, antioxidant mechanisms, and anti-inflammatory responses (Qian et al., 2018; Xue et al., 2018). Over ten neural bridge devices have progressed to the clinical application stage, utilizing 3D printing technology, hydrogel technology, and nanotechnology (Gu, Ding, and Williams, 2014; Chen et al., 2019). Currently, chitosan, which is commonly used to construct nerve conduits, is the most common natural polymer in polysaccharide chitin and is widely studied and used as a nerve scaffold (Parveen and Sahoo, 2011; Hilitanu et al., 2022). Chitosan-containing complexes can support axonal regeneration and reduce the amount of scar tissue and neuroma, with good biocompatibility, antimicrobial activity, extraordinary degradability, and no cytotoxicity (Khan et al., 2021). The material possesses chemical and physical properties that facilitate the reproduction of the physiological makeup observed in peripheral neurons (Wang, Zhao, et al., 2016). The neurotrophic factor BDNF, which is often loaded in nerve conduits, has biological functions of neuroprotection and growth stimulation (Gill et al., 2003; Nagahara et al., 2009; Bertram et al., 2010; Rejdak et al., 2023). BDNF has a high clearance rate and an extremely short plasma half-life, which attenuates nerve cell death, promotes angiogenesis, increases synaptic plasticity, and reduces neuroinflammation (Schabitz et al., 2007; Liu et al., 2020). The process under consideration contributes significantly to the preservation of neuronal viability, even in instances where transient intercellular connections are absent (Colucci-D'Amato et al., 2020; Manoukian et al., 2021). This process significantly contributes to the development and production of myelin sheaths in peripheral nerves (Xiao et al., 2010; Wilhelm et al., 2012; Numakawa, Odaka, and Adachi, 2018; Langhnoja, Buch, and Pillai, 2021).

**FIGURE 1:**
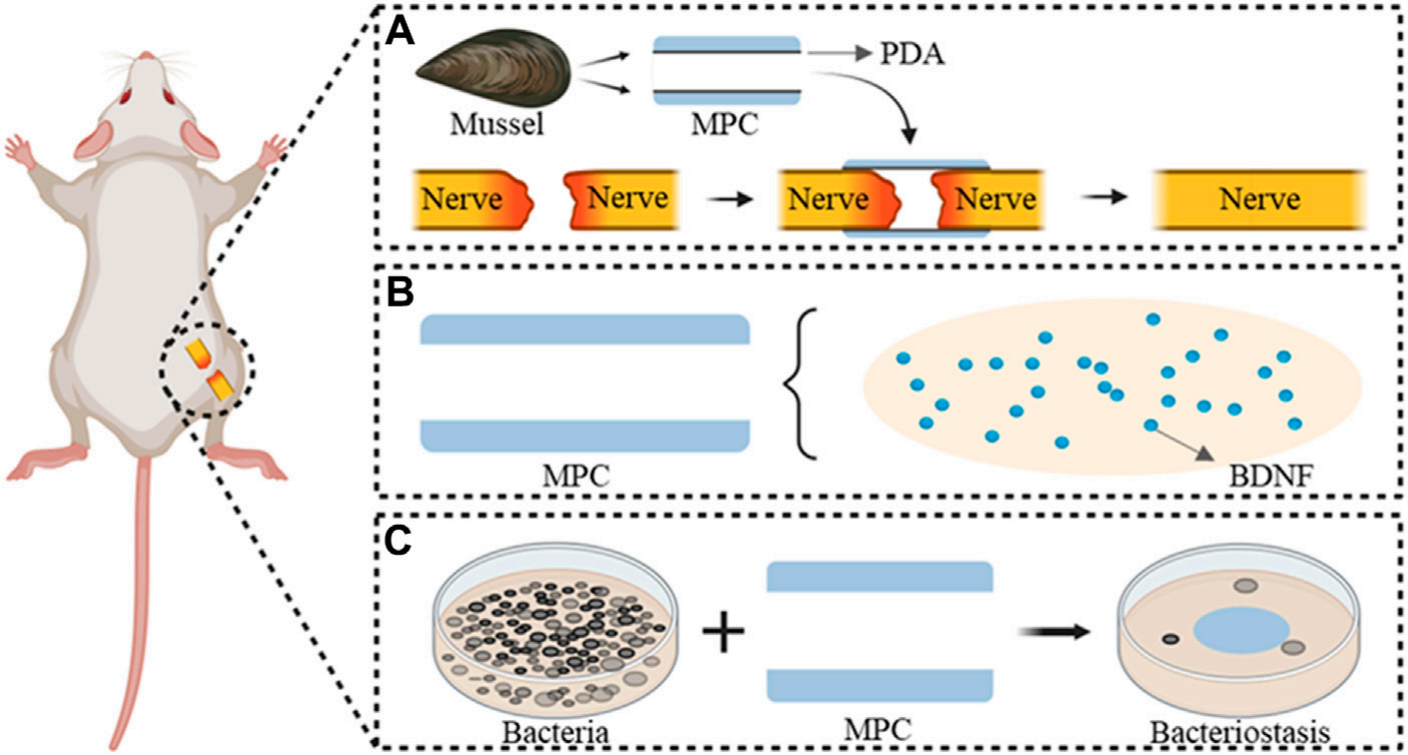
MPC@BDNF Mechanisms and characteristics of the repair of peripheral nerve injury. **(A)** Suture-free bridging of MPC fabricated from a mussel-mimicking polymer in the peripheral nerve injury area. **(B)** BDNF pattern diagram of MPC internal loading. **(C)** Antimicrobial properties of the implant material MPC@BDNF.

However, the conventional nerve conduit is characterized by its hollow construction, although its independent application fails to satisfy the demands for prompt regeneration and clinical restoration of function in instances of peripheral nerve injury. Therefore, identification of an appropriate nerve conduit via tissue engineering, establishment of an optimal regenerative microenvironment, replication of the natural extracellular matrix, and incorporation of nerve growth mediators are imperative. These measures aim to facilitate nerve regeneration and offer a more precise alternative to conventional nerve transplantation. This approach functions as a “bridge” to effectively heal large nerve injuries (Singh et al., 2018; Lu et al., 2023). It is desirable to incorporate various guidance cues into a singular conduit during the development of a neural conduit that possesses suitable mechanical strength to prevent lumen collapse and suture pullout. This integration aims to facilitate the migration of endogenous cells and the regeneration and extension of axons, ultimately leading to nerve regeneration and the restoration of functionality. The aim of this study was to inject polydopamine (PDA) into prepared mimetic mussel polymer conduits (MPC) with adhesive properties that allow bridging of injured peripheral nerves without sutures (Figure 1A). Thus, the operation time was reduced, BDNF was loaded (Figure 1B), the microenvironment of the injured site was improved, and nerve regeneration was accelerated. The antibacterial properties of the implanted material MPC@BDNF were assessed to confirm its antibacterial efficacy (Figure 1C). In a rat model of 15 mm sciatic nerve injury, the aim of this study was to evaluate the therapeutic efficacy of a specially designed nerve conduit.

## 2 Materials and methods

### 2.1 MPC and MPC@BDNF fabrication

**FIGURE 2:**
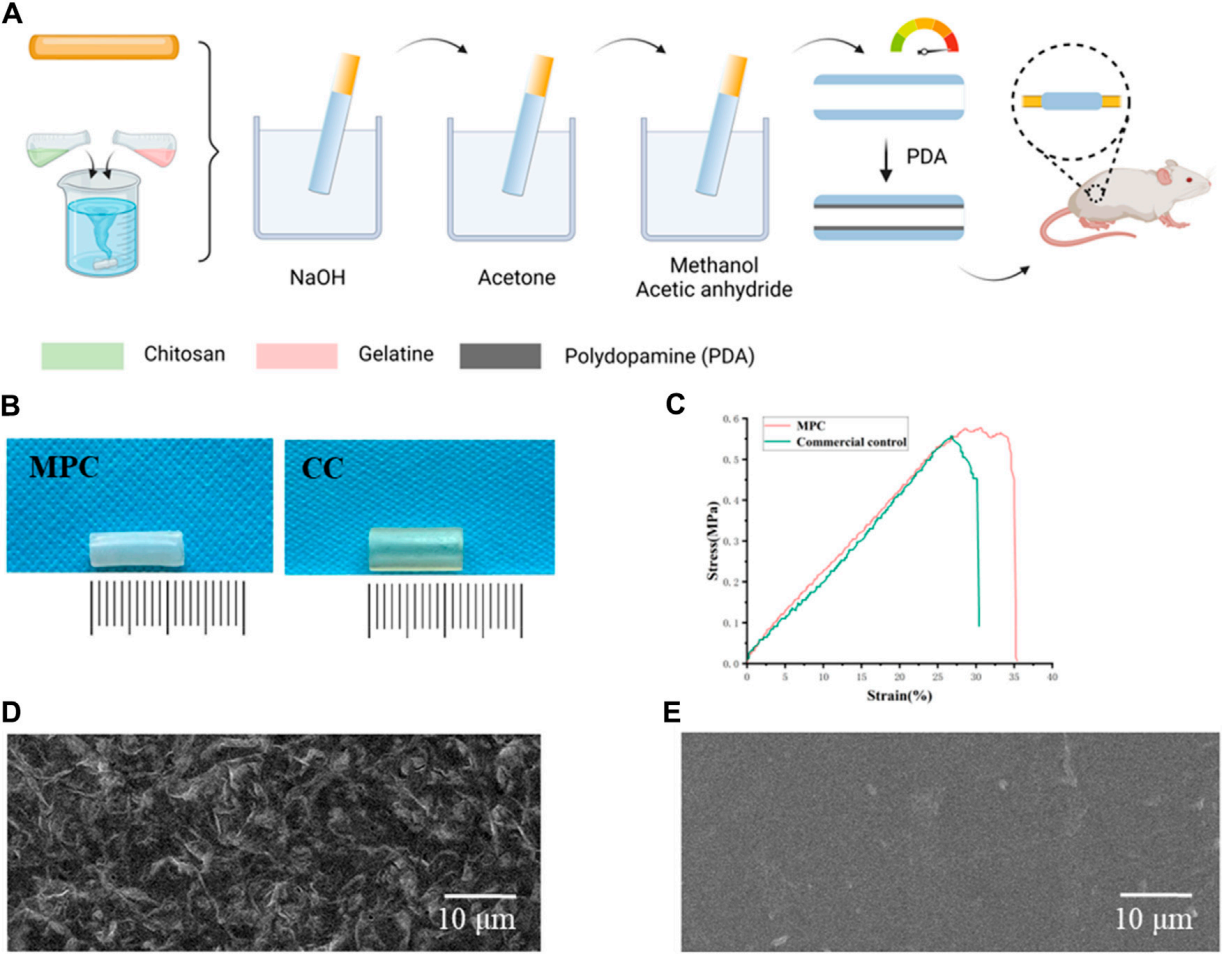
Characterization and preparation of MPC. **(A)** Schematic diagram of MPC fabrication. **(B)** Images of the MPC and CC. **(C)** Stress‒strain curves of MPC and CC. **(D)** Electron micrographs of the rough inner surface of the MPC. **(E)** Electron micrographs of the smooth inner surface of the CC.

Chitin has a specific 164.2 kDa (kDa), a viscosity of 1,500 cP (CP), and a degree of deacetylation exceeding 80%. The chitin was sourced from Tongzhou, Beijing, a subsidiary company of the ninth Hospital of the Ministry of Industry. A total of 140 g of chitin and 3,500 mL of a 2% acetic acid solution were utilized in the experimental procedure. After stirring for 2 h, a 4% chitosan solution was obtained. 14 g of gelatin (Sigma) and 875 g of distilled water were dissolved at 70°C and stirred for 1 h to promote faster and more uniform dissolution of the chitin, and 1.6% gelatin was obtained. After the temperature decreased, 4% chitosan and 1.6% gelatin were mixed to achieve a viscosity of 90,000 cP. The samples were passed through a 250 mesh metal filter, filtered and allowed to stand for 1–2 days to defoam. The nerve conduits were prepared by manufacturing molds with different calibres. After the nerve conduits were made, they were put into 5% NaOH, removed, and placed in water for 24 h to clean them neutral. The samples were dehydrated with acetone for 2 min and then allowed to dry for 10 min. A total of 250 mL of methanol and 250 mL of acetic anhydride were added for 10 min, and the solution was removed when the nerve cannula was clear (Figure 2A). The PDA was adhered to the inside of the nerve conduits to complete the MPC, which was used when needed.

To prepare MPC@BDNF, BDNF (11166-BD, R&D Systems, United States) at a concentration of 30 ng/μL was mixed with 4% chitosan and made into MPC@BDNF tubes as described above.

#### 2.1.1 Scanning electron microscopy (SEM)

The specimens were observed with a scanning electron microscope (SEM) (TESCAN MIRA LMS, Czech Republic) to observe the structure of the monolayer phase change. Initially, the MPC was placed in a −20°C refrigerator for 4 h, completely cooled, and then transferred to a Vacuum Freeze Dryer (SCIENTZ-10N, Ningbo, China) to start freeze-drying for 36 h. A stratum of conductive material was applied to the provided samples after they had been frozen and dried using the cryofixation procedure. The SEM sample stage was used to mount the prepared samples, which were subsequently attached to a sample holder. To establish high vacuum conditions before the SEM observations, the SEM chamber was evacuated. Upon careful analysis of the sample surface and the interior graphs of the MPC and commercial control (CC), the sample surface was observed and photographed using an electron beam at an accelerating voltage of 3 kV at 1,000 × magnification.

### 2.2 Mechanical tests

Tensile strength is the maximum tensile force that a material can withstand per unit area, and is usually used to measure the strength and toughness of the material. It is the ability of a material to resist breaking when subjected to tension or tension. It is of great significance for the selection and application of materials. We test and analyze the tensile strength of materials to better understand MPC and CC mechanical properties. The material had a width of 3 mm and a thickness of 1 mm, and the mechanical characteristics of the MPC and CC were measured by performing three successive tensile tests on each sample with a universal testing instrument (MARK-10, New York, United States). The maximum force applied at the point of failure is recorded, and the tensile strength is calculated by dividing this force by the cross-sectional area of the sample. The stress‒strain curve was examined, and the percentage of fracture strain was determined.

### 2.3 Adhesion test

Sutureless surgery is crucial for repairing peripheral nerves, which is based on tissue adhesion ability. We attached the MPC to a slide on which the kidney, liver, lung, heart, and muscle were located on the opposite side, pressed the tissue vigorously for 6 s, and then inverted 180° for imaging. We created 2.5 cm wide adhesion specimens, affixed MPC to various biological tissues, and used a mechanical tester (ASTM F2256) to determine the interface toughness. After the adhesion of MPC for 20 min, the 180° peel strength test was conducted by subjecting the samples, with a stretching rate of 18 mm/min and the temperature range of 180°C–300°C. Once the peeling process reaches a stable state, the corresponding measured force also stabilizes. At this point, the quantification of the interface toughness can be achieved by multiplying the applied force during the peeling process by the width of the tissue sample.

### 2.4 *In vitro* cell viability assays

The biocompatibility of the MPC was evaluated using live/dead cell staining, and CC was used as a control for comparison with the MPC group. Prefabricated nerve conduits composed of acetylated chitosan served as the control group (Beijing Huifukang Medical Technology Co., Ltd., 20,213,130,298). The RSC96 cell line (ATCC, CRL-2764) was maintained in culture at 37°C in a carbon dioxide incubator with a 5% CO_2_ atmosphere. The cells were then seeded into sterilized multiwell plates after two to three passages. The RSC96 cells were cultured in separate nerve tubes for 1, 3, or 7 days. Following the instructions provided by the Calcein-AM/PI labeling kit (Beijing Solarbio, Beijing, China, CA1630-500T), 200 μL of the cells were stained for 6 min. The fluorescence images recorded by an inverted fluorescence microscope (Zeiss) were used to assess the growth and viability of the RSC96 cells. In these photos, live cells were stained green, while dead cells were dyed red.

### 2.5 Staining of the cytoplasm

To demonstrate that CC, MPC and MPC@BDNF promote cell proliferation and regeneration, we cultured RSC96 cells with MPC@BDNF, MPC and CC in culture medium at 37°C. Evidence of regenerative proliferation of RSC96 cells was observed by staining analysis with a Bestbio Biologics erythrocyte chromatin kit (BB-441256).

### 2.6 Antimicrobial tests

The antibacterial effect of materials is essential for successful surgical repair of PNI. We used an antibacterial ring assay to verify the antibacterial activity of MPC. A bacterial culture was prepared by growing the bacteria on AGAR plates until full growth was achieved (*Staphylococcus aureus*, ATCC 29213; *Escherichia coli*, ATCC 25922). A cylindrical MPC with a diameter of 0.5 cm was irradiated using UV light for 30 min prior to antibacterial studies. MPC was seeded on the surface of a solid AGAR plate uniformly coated with bacterial suspension and determined to be in contact with the bacterial culture. The plates were gently pressed with sterile tweezers to allow adequate contact of the sample with the medium and subjected to incubation for 24 h at 37°C in a constant temperature warmer. Upon completion of the culture, images were taken with an ordinary camera, and the inhibition zone around the Muller-Hinton AGAR plate was measured with a scale caliper. The antibacterial efficacy of the nerve conduit was assessed based on the size of the inhibitory ring, where a larger diameter indicated a greater antibacterial efficiency of the MPC. An inhibition zone or no inhibition zone with a smaller diameter indicated weak or no antibacterial efficiency of the MPC.

### 2.7 *In vivo* investigation

Research on the processes involved in peripheral nerve regeneration and repair often uses an animal model of sciatic nerve injury in rats. Female SD rats (Weitong Lihua Vitar River Laboratory Animal Science and Technology Co., Ltd., Beijing, China) that were 4–6 weeks old and weighed 200–220 g were anesthetized with 5% isoflurane (Zhongmu Beicang Pharmaceutical Co., Ltd., Beijing, China). Following the administration of gas anesthetics, shaving and cleaning procedures were applied, and a minor surgical incision was made on the outer skin of the thigh to expose the right sciatic nerve. Cutting across the sciatic nerve resulted in a 15 mm injury, hence establishing the standard animal model for sciatic nerve damage. The rats were divided into three cohorts, with each cohort including five rats, according to the repair materials employed: the MPC group, the MPC@BDNF group, and the CC group. The rats were given a razor to depilate its hindlimb, and then iodophor was used to clean the skin. Subsequently, three nerve injury distances were simulated based on the exposure of the sciatic nerve. In the MPC group and MPC@BDNF group, adhesive repair was used to repair the nerve, while in the CC group, traditional suture and chitosan nerve repair catheters were used to repair the nerve, and all wounds were closed after careful disinfection. After surgery, all rats were given adequate food and water and allowed to move freely, and vital signs were monitored. The regenerated sciatic nerve was observed and evaluated again 12 weeks after surgery. The Laboratory Animal Ethical and Welfare Committee of Shandong University Cheeloo College of Medicine granted approval (Approval No. 23027) for the experimental programs involving mice. The animal care procedures conformed to the Chinese criteria for the Ethical Assessment of Experimental Animals for the Welfare of Animals (GB/T 35892–2018), and the reporting of animal data followed the ARRIVE 2.0 criteria.

### 2.8 Immunofluorescence

To illustrate the impact of MPC@BDNF and MPC on the facilitation of axonal regeneration, a total of five rats in each experimental group were euthanized using excessive carbon dioxide at a filling rate ranging from 30% to 70% per minute. This procedure was conducted 12 weeks postsurgery. The nerve transplants were procured, preserved, and then mounted on slides using a cryostat microtome for immunofluorescence staining with NF200, S100, and DAPI. The degree of axonal regeneration was assessed by analyzing the extent and dispersion of NF200 staining, whereas the existence and distribution of myelin sheaths were visualized using S100 staining. And the thickness of section on which the immunostaining was 5 mm.

### 2.9 Morphological analysis of regenerating axons

To assess the dimensions of the regenerated myelin, specifically its diameter and thickness, the specimen slices were subjected to a fixation process using a 2.5% glutaraldehyde solution (Millipore, Sigma) at a temperature of 2°C for 2 h. Following fixation, the specimens were immersed in a clear epoxy resin and later divided into thin sections measuring 700 nm (referred to as semithin sections) and 70 nm (referred to as ultrathin sections). Subsequently, the aforementioned sections were subjected to staining procedures, which included citrate of lead and uranyl acetate. The tiny sections were transferred onto a mesh designed for transmission electron microscopy (TEM). A TEM device made in Amsterdam. The Philips Netherlands was then used for examining the cells. To determine the newly generated myelin thickness, fiber diameter, and density of regenerated axons, ImageJ 10.6 software, developed by the National Institute of Health in Bethesda, MD, was used. This analysis was performed on each set of five randomly selected TEM images.

### 2.10 Gastrocnemius muscle characterization

Gastrocnemius muscle specimens from the hind limbs of SD rats were subjected to fixation using a 4% paraformaldehyde solution at 4°C for 24 h. Following fixation, the specimens were embedded in paraffin, and cross-sectional slices with a thickness of 5 μm were produced. The effects of MPC@BDNF, MPC, and CC on promoting functional recovery of injured peripheral nerves were tested with a Masson trichrome staining kit (Solarbio Technology Co., Ltd., Beijing, China). The purpose of this investigation was to determine whether collagen fibers (dyed blue), muscle fibers (stained red), red blood cells (stained red), or nuclei (stained black or dark blue) were present in the sample. The quantification and analysis of the color and spatial arrangement of these components can be performed using ImageJ 10.6 software, which enables the identification of various tissue types and pathological alterations. To evaluate the progress in functional recovery of the organ that was targeted, the gastrocnemius muscle was measured to determine its diameter and density subsequent to peripheral nerve reconstruction.

### 2.11 Gait analysis

Evaluating the recovery of motor abilities subsequent to the rehabilitation of the injured sciatic nerve in rats is a key process for validating our findings, and the sciatic nerve function index (SFI) is an employed approach for assessing the progress of sciatic nerve regeneration in rats. Following the rats’ acclimation to the planned orbital environment, we employed the CatWalk XT 10.6 gait analysis system (Noldus, Wageningen, Netherlands) to evaluate various groups of rats 12 weeks postsurgery. Three investigators, who were unaware of the experimental groups, recorded the walking trajectory, such as the paw print area and paw strength, through a high-speed video camera (Noldus). The SFI for every rat was assessed and evaluated using Bain’s formula, which is expressed as follows: SFI = 109.5*(ETS - NTS)/NTS +13.3*(EIT - NIT)/NIT −38.3*(EPL-NPL)/NPL −8.8. In the given context, the variables are defined as follows: E denotes the side that has sustained an injury, N represents the unaffected side, TS signifies the length of the claw (measured from the first to the fifth toe), PL denotes the length of the claw (located between the third finger and the heel), and IT represents the spread of the middle toe (the measurement is determined by the spatial separation between the second and fourth digits of the foot.). An SFI of approximately 0 signifies the presence of normal nerve function, while an index of approximately −100 signifies the occurrence of full nerve injury.

### 2.12 Statistical analysis

The statistical analysis of all values obtained in this study was conducted using the Graph Prism program version 10.0 (GraphPad Software, Inc., La Jolla, CA, United States). The results are presented as the mean ± standard error of the mean (SEM). The various categories were subjected to one-way analysis of variance (ANOVA) to assess and compare their differences. Once the assumption of homogeneity of variance was met, Tukey’s *post hoc* test was conducted. Dunnett’s *post hoc* test was employed to assess heterogeneity. In all the statistical studies conducted, the threshold for determining statistical significance was set at a significance level of *p* < 0.05.

## 3 Results

### 3.1 Mechanical properties

As shown, the prepared MPC was milky white (Figure 2B) and tightly packed into bundles. We characterized the inner surface structure of the MPC and CC by electron microscopy (Figures 2D,E), and low-power images showed a uniform fiber size. Higher magnification images show the surface details of the MPC. The observed characteristics of the nerve conduit include a uniform distribution and a rough inner surface that is suitable for the loading of cytokines. Additionally, it is imperative for these conduits to possess adequate mechanical strength to effectively support the directed elongation of nerve axons when used as implantable devices. Accordingly, the mechanical information of the MPC and CC under the load of a universal material testing machine was tested. The stress‒strain curve was subsequently plotted in a customary manner (Figure 2C). The experimental findings indicated that the MPC material exhibited a tensile strength of approximately 0.59 MPa and a maximum tensile strain of 36%. In contrast, the CC material demonstrated a tensile strength of approximately 0.54 MPa and a maximum tensile strain of 30%. MPC shows better mechanical toughness and tensile properties than CC, which can address the fundamental requirements of materials for peripheral nerve healing.

### 3.2 Adhesion characteristics

**FIGURE 3:**
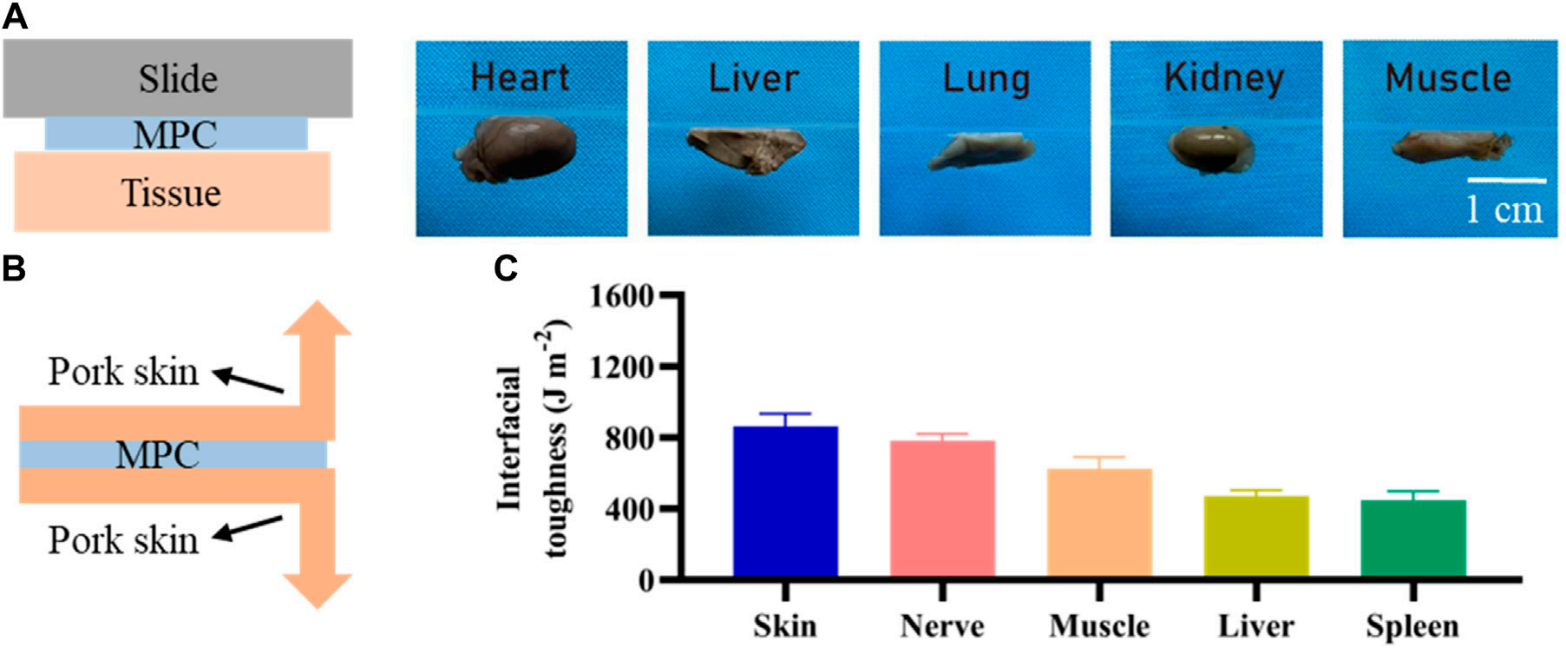
Adhesion of MPC. **(A)** Adhesion of the MPC to different organs and tissues. **(B)** Schematic diagram of the 180° stripping experiment. **(C)** Toughness of MPC tissue/organ adhesion contact.

The presence of robust adhesions is of paramount significance in the context of nerve injury regeneration, so we investigated the adhesion of the MPC. As shown in Figure 3A, MPC can firmly and effectively adhere to the surface of different biological tissues, such as the gastrocnemius muscle, heart, liver, kidney, and lung, when lightly pressed for 6 s. Therefore, the MPC can bridge the ends of the PNI tightly and stably without sutures, forming a dense gap and prolonging the duration of BDNF factor loading. Compared with traditional surgical repair, which needs to protect the nerve from movement. The utilization of MPC has been found to effectively mitigate the challenges and time constraints associated with surgical operations. Furthermore, MPC circumvents the potential occurrence of secondary injuries resulting from the use of sutures and suture needles while also minimizing the likelihood of postoperative pain caused by suturing. Therefore, it can be inferred that MPC exhibits significant promise for *in vivo* application. Moreover, the interfacial toughness was evaluated through experimental testing after 20 min of adhesion between the MPC and different biological tissues via 180° stripping experiments. As shown in Figure 3C, skin, nerve and muscle showed the highest interfacial toughness with MPC, all of which were greater than 600 J m^−2^, while the interfacial toughness of liver and spleen were less than 500 J m^−2^, indicating that MPC and nerve could adhere better *in vivo*.

### 3.3 Assessment of compatibility *in vitro*

**FIGURE 4:**
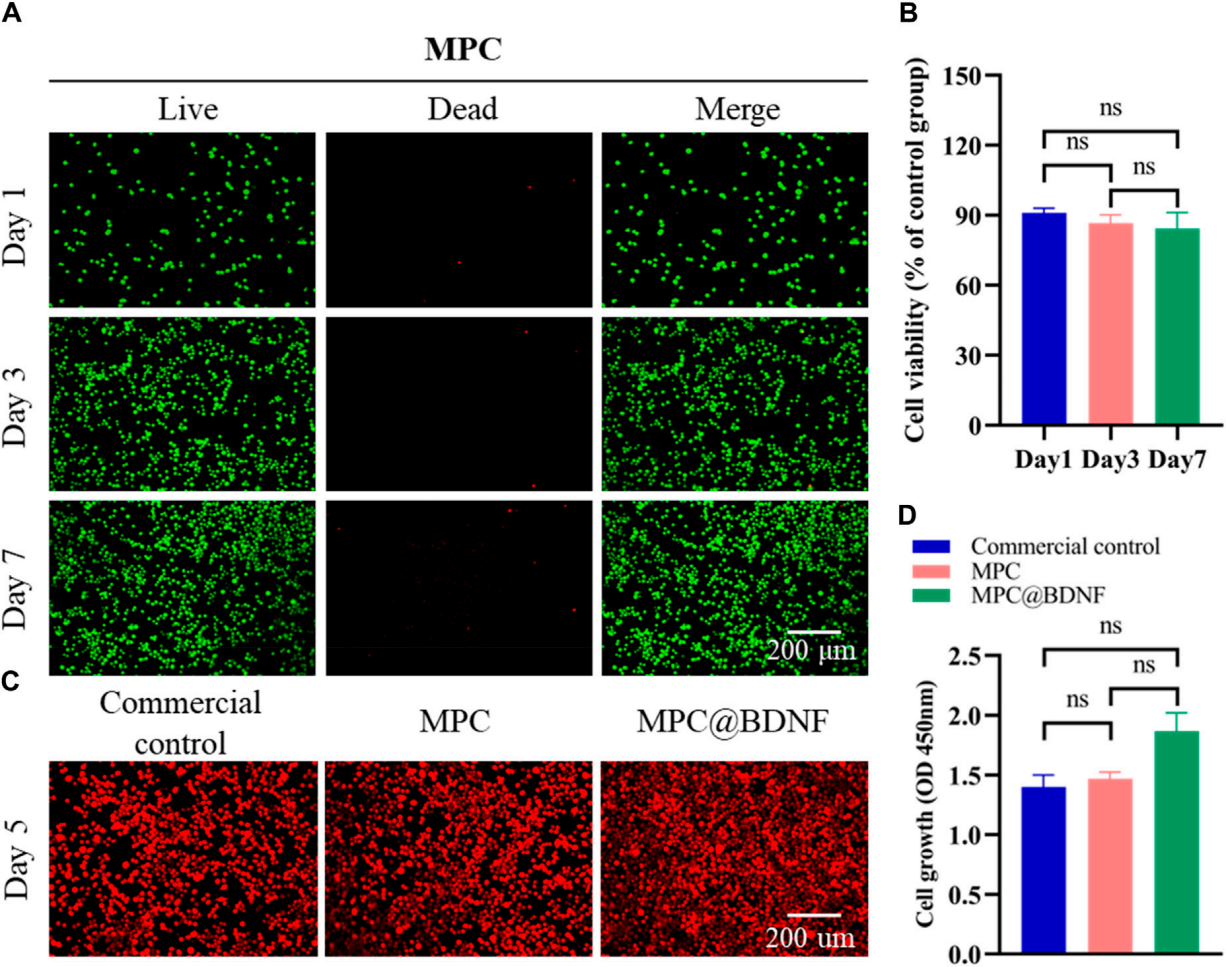
*In vitro* compatibility of MPC. **(A)** A calcein-AM/PI staining kit was used to visualize RSC96 cell viability/death in MPC-cocultured cells. **(B)** CCK-8 assays were used to determine the effect of RSC96 cells on MPC activity after 1, 3, and 7 days (ns: no significant difference). **(C)** Number of RSC96 cells in the CC, MPC, and MPC@BDNF groups on the fifth day. **(D)** Cell optical density (450 nm) values of cell proliferation among the CC, MPC, and MPC@BDNF groups (ns: no significant difference).

The assessment of the favorable *in vitro* compatibility of MPC using a cytotoxicity assay is important for determining its suitability for regeneration of PNI. Live/dead staining and CCK-8 assays are commonly employed for assessing the viability of RSC96 cells. In the initial stage of the cell research, RSC96 cells were cultured on flat smooth MPC sheets and subjected to staining procedures on days 1, 3, and 7. A calcein-AM/PI staining kit showed that RSC96 cells density (labeled with green cells) significantly increased over time after 1, 3 and 7 days. The quantity of deceased cells, namely, those identified as red cells, decreased. Additionally, the RSC96 cells demonstrated a sustained high level of proliferative activity for 7 days and disseminated across the entirety of the visual field (Figure 4A). Moreover, there was no statistically significant difference in the viability of the RSC96 cells that were implanted with MPC between days 1 and 3, between days 3 and 7, or between days 1, 3, and 7 (Figure 4B). MPC exhibits similarities to mature nerve conduits and demonstrates remarkable biocompatibility, rendering it a promising contender for future applications in peripheral nerve injury healing.

### 3.4 Cell proliferation

To ascertain the impacts of CC, MPC and MPC@BDNF on the propagation of RSC96 cells, we seeded the cells in the above three groups and calculated the cell proliferation on the fifth day. According to the data presented in Figure 4C, the quantity of RSC96 cells was greatest in the MPC@BDNF group, followed by the MPC group, and the lowest in the CC group. The optical density (450 nm) of cell proliferation on day 5 did not significantly differ among the CC, MPC, and MPC@BDNF groups (Figure 4D).

### 3.5 Antimicrobial properties

**FIGURE 5:**
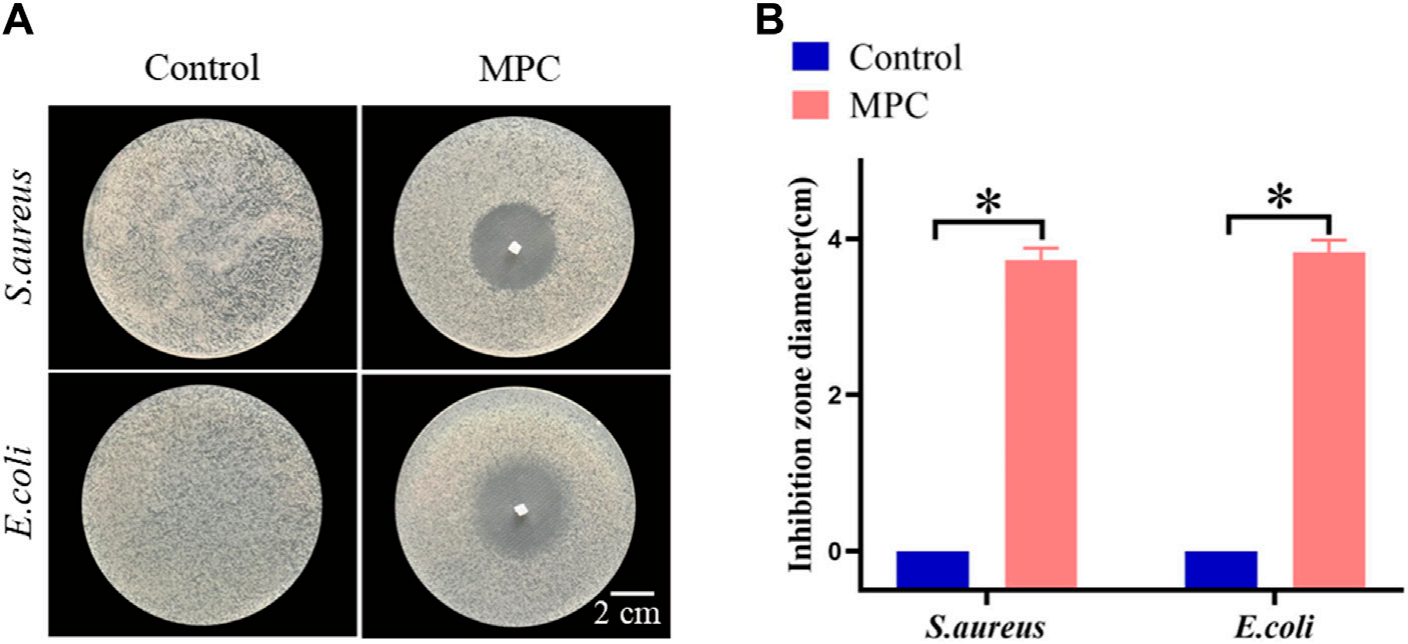
The antibacterial properties of MPC. **(A)** The bacteriostasis ring test was used to determine how well MPC killed *Staphylococcus aureus* and *Escherichia coli* bacteria. **(B)** Statistics of the antibacterial effects of MPC against *Escherichia coli* and *Staphylococcus aureus* (*p < 0.05).

As a material for implantable repair of PNI, antimicrobial activity is of paramount importance. Since MPC is made from chitosan, it also has excellent antimicrobial activity. In this study, the efficacy of MPC in preventing the growth of *S. aureus* and *E. coli* was evaluated through the use of the antibacterial ring method. The extent of inhibition observed, as indicated by the diameter of the inhibition zone, served as an indicator of the antibacterial effectiveness of the MPC. The findings indicated that the application of MPC on Petri plates coated with *S. aureus* and *E. coli* resulted in the creation of distinct antibacterial rings (Figure 5A). Hence, it can be inferred that the utilization of MPC has the potential to effectively mitigate the risk of infection resulting from PNI repair while concurrently minimizing the incidence of postoperative problems. Furthermore, the antibacterial effects of MPC and the control on *S. aureus* and *E. coli* were not statistically significant (Figure 5B).

### 3.6 *In vivo* applicability and nerve regrowth applicability

To assess the velocity and effectiveness of MPC therapy in facilitating the regeneration of PNI in rats, the distal regenerated sciatic nerve was surgically removed at week 12 post-repair. This was done to enable subsequent immunofluorescence and TEM analysis. We measured the number of axons and myelin sheaths in the nerve tissue that had regenerated. This helped us determine how much peripheral nerve regeneration occurred.

**FIGURE 6:**
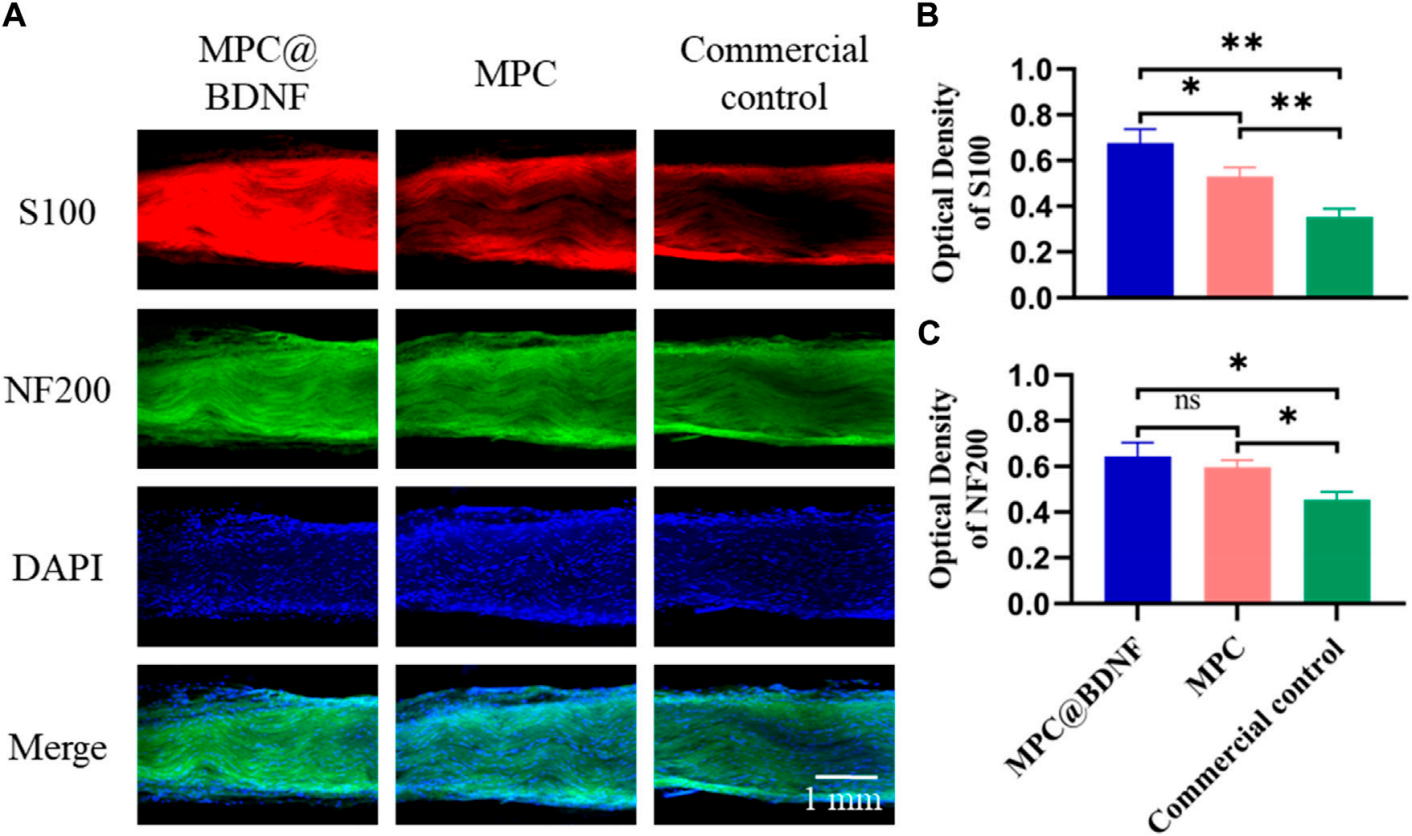
MPC@BDNF, MPC, and CC promoted histological characterization of lost peripheral nerve regeneration. **(A)** The schematic representation displays the immunofluorescence labeling of the sciatic nerve using S100, NF200, DAPI, and Merge. **(B and C)** Statistical analysis of S100 and NF200 immunofluorescence staining in each experimental group (ns: no significant difference, *p < 0.05, **p < 0.01).

Immunofluorescence of the central cross sections of nerve grafts obtained from rats in each experimental group revealed the presence of green fluorescence (NF200), which is indicative of axons; red fluorescence (S100), which is indicative of Schwann cells (SCs); and blue fluorescence (DAPI), which is indicative of nuclei (Figure 6A). In this study, a set of typical pictures was carefully chosen for observation. The number of regenerating axons and the visual density of the myelin sheath were quantified using ImageJ software and S100 and NF200 immunofluorescence labeling. The results of S100 immunofluorescence analysis revealed that both the MPC@BDNF group and the MPC group exhibited a greater density of myelin sheaths than did the CC group. Additionally, the MPC@BDNF group demonstrated a greater number of remyelination events than both the MPC group and the CC group. Furthermore, the MPC group exhibited a greater number of remyelination events than did the CC group. A statistically significant difference in the extent of remyelination was observed across the CC, MPC and MPC@BDNF groups (Figure 6B). The results obtained from NF200 immunofluorescence analysis revealed that the density of regenerated axons in both the MPC@BDNF group and the MPC group was significantly greater than that in the CC group. There was no statistically significant difference in the quantity of regenerated axons in the distal nerve between the MPC@BDNF group and the MPC group. However, statistically significant differences in the number of regenerated axons were detected between the MPC@BDNF group and the CC group and between the MPC group and the CC group (Figure 6C).

**FIGURE 7:**
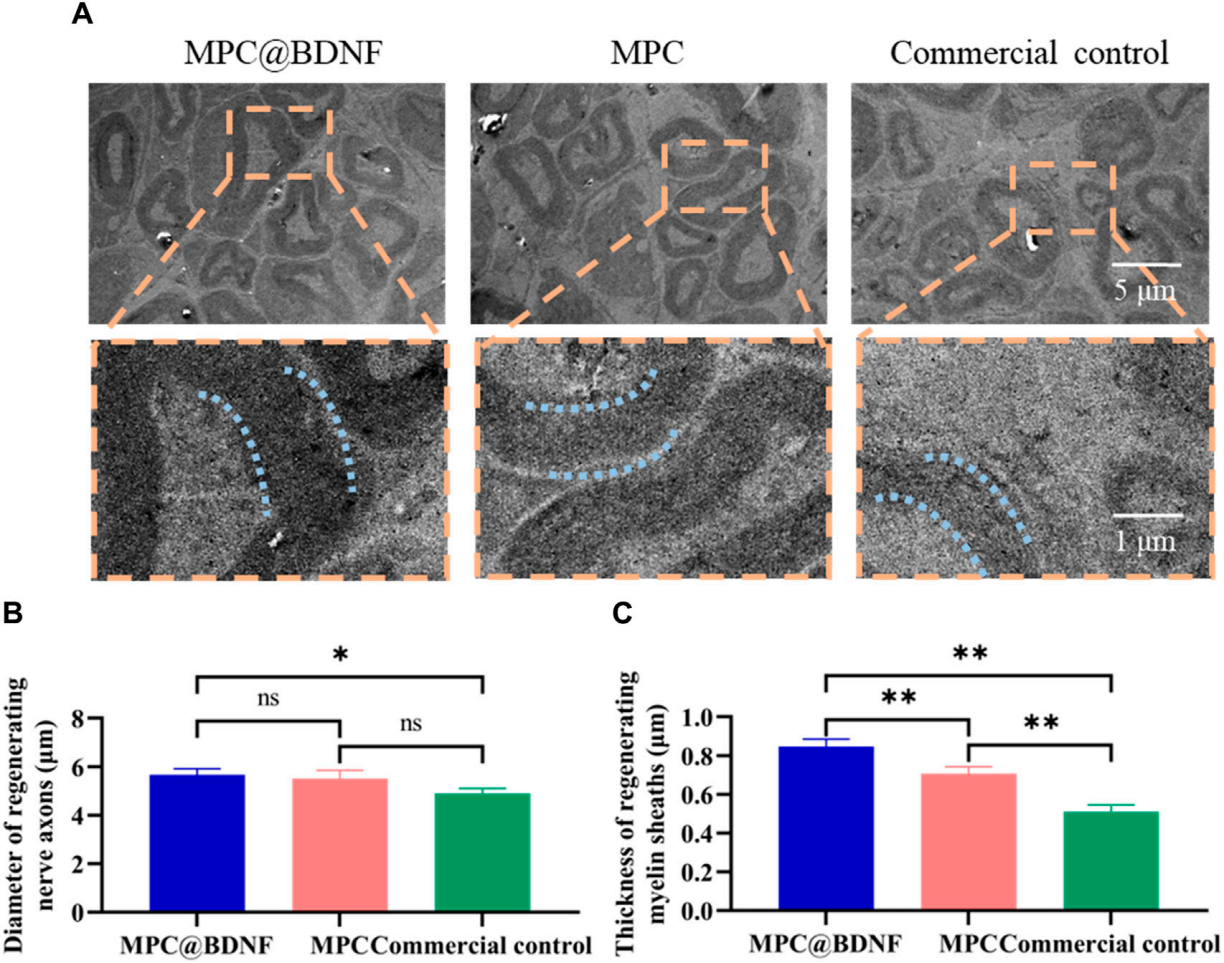
MPC@BDNF, MPC, and CC promoted histological characterization of lost peripheral nerve regeneration. **(A)** A transmission electron microscope was used to observe cross-sections of nerve myelin sheaths that grew back in the MPC@BDNF, MPC, and CC groups. **(B)** Comparison of the sizes of nerve myelin sheaths that grew back between groups (ns: no significant difference, *p < 0.05). **(C)** Statistical analysis of regenerating nerve myelin sheath thickness in each group (**p < 0.01).

The presence of well-developed myelin sheaths with a rounded shape and substantial thickness has been correlated with enhanced neurological function. To investigate this relationship, we employed electron microscopy to examine the morphological characteristics, specifically the dimensions, namely, the diameter and thickness, of regenerating myelin sheaths (Figure 7A). The results of our study demonstrated that the diameter of the myelin sheath was significantly greater in both the MPC@BDNF and MPC groups than in the CC group. No statistically significant difference was found between the MPC@BDNF and MPC groups. However, a significant difference was detected among the MPC@BDNF and CC groups. Additionally, there was no statistically significant difference among the groups assigned to the MPC and CC (Figure 7B). Moreover, according to the myelin sheath thickness, the MPC@BDNF group exhibited the greatest myelin sheath thickness, followed by the MPC and CC groups. Notably, statistically significant disparities in myelin sheath thickness were observed among these three groups (Figure 7C).

The findings indicated successful anastomosis of the severed nerve terminals across all the experimental groups, with varying degrees of healing observed in the regenerated nerves. In comparison to CC, the administration of MPC@BDNF had a substantial impact on promoting the regrowth of both the diameter and the thickness of the myelin sheath in peripheral nerves.

### 3.7 Evaluation of neurological function

**FIGURE 8:**
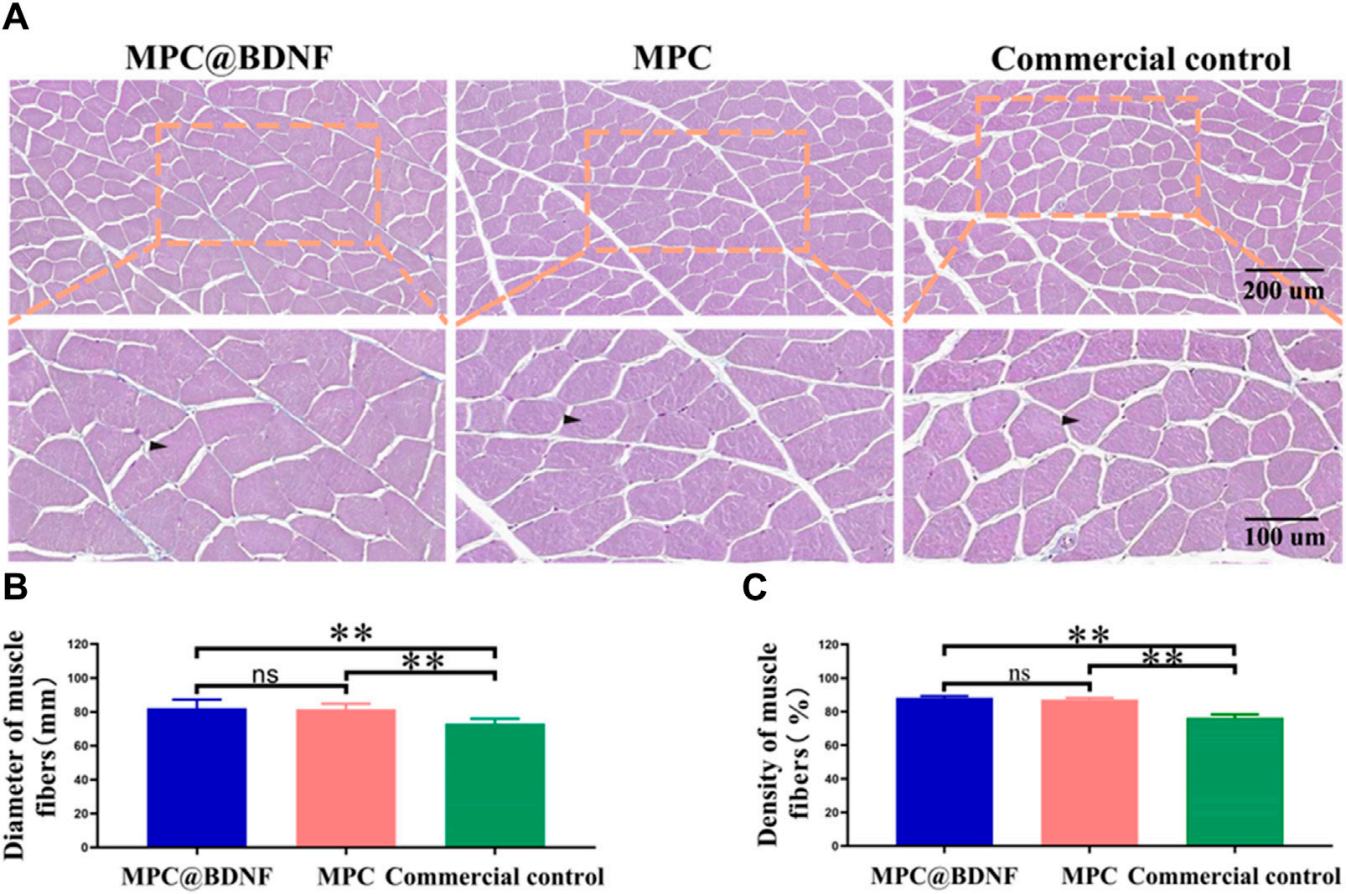
Recovery of muscle tissue in rats by CC, MPC, and MPC@BDNF. **(A)** The gastrocnemius muscle in each group was subjected to Masson trichrome staining. **(B and C)** Statistical analysis of muscle fiber diameter and muscle fiber density among the groups (ns: no significant difference, *p < 0.05, **p < 0.01).

Histological studies and behavioral tests are commonly employed in the evaluation of nerve regeneration in rats subsequent to PNI. The examination of the function and structure of the gastrocnemius muscle, which serves as the target organ innervated by the sciatic nerve, can provide insights into the extent of nerve regeneration. At 12 weeks after nerve transplantation, we evaluated muscle status by observing muscle fiber diameter and muscle fiber density in muscle samples by Masson trichrome staining. Representative images of the gastrocnemius muscles of the MPC@BDNF, MPC, and CC groups are shown (Figure 8A). The muscle fiber width and muscle fiber density of the MPC@BDNF and the MPC were significantly greater than those of the CC. The muscle fiber density and muscle cross-sectional area of both the MPC@BDNF group and the MPC group were significantly different from those of the CC group. However, no statistically significant differences were detected in terms of muscle fiber diameter or muscle fiber density between the MPC@BDNF group and the MPC group (Figures 8B,C). MPC and MPC@BDNF have demonstrated enhanced efficacy in the preservation of optimal muscle mass and the establishment of a robust foundation for functional rehabilitation following reinnervation. Furthermore, hematoxylin and eosin (HE) staining of critical organs, such as the liver, kidney, heart, lung, spleen, and gastrocnemius muscle, revealed no notable pathological alterations. These findings are visually represented in Figures 1–5 and may be found in the accompanying Supplementary Material.

**FIGURE 9:**
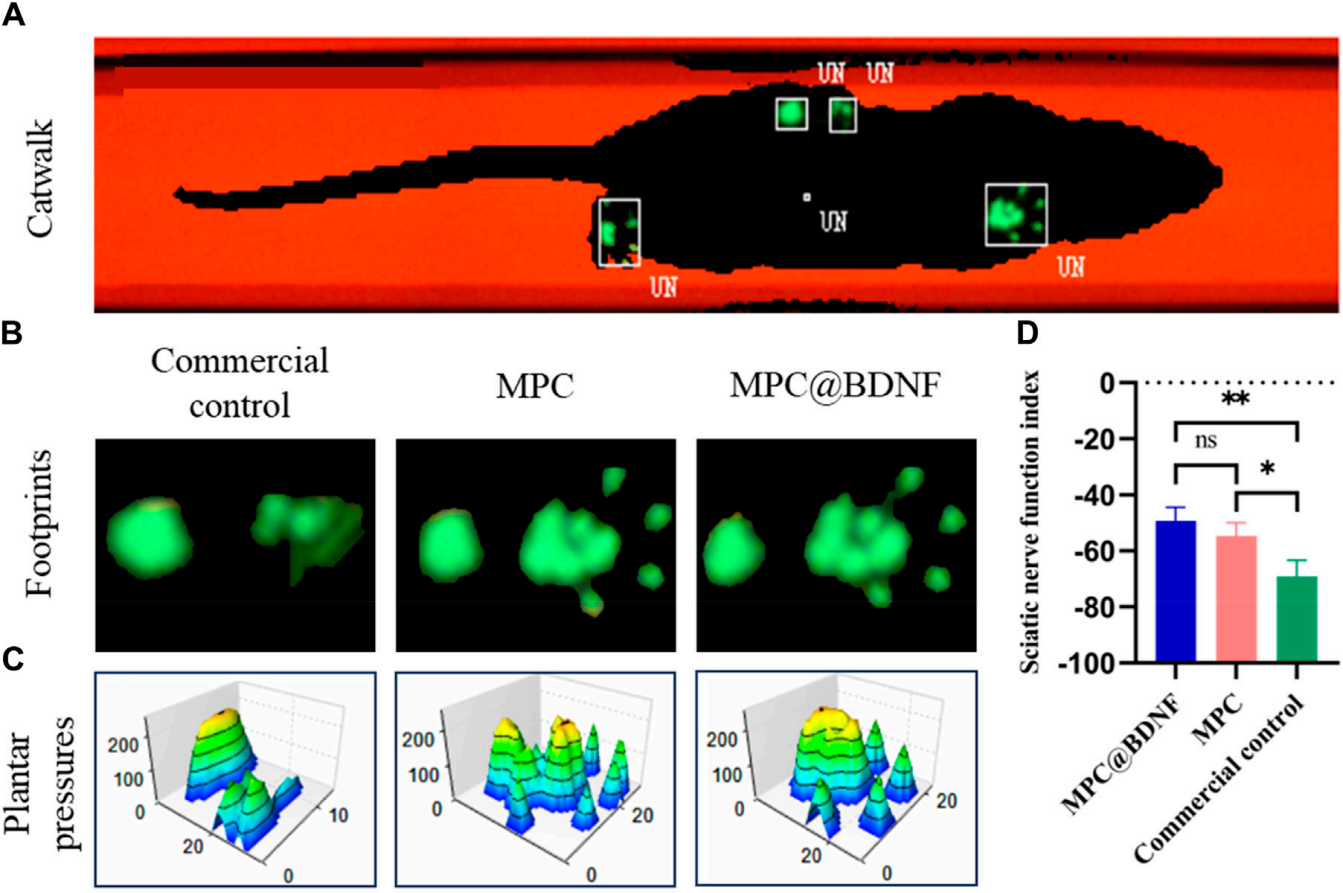
The functional recovery of CC, MPC, and MPC@BDNF in rats with sciatic nerve injury. **(A)** A diagram of a typical rat’s footprint 12 weeks after surgery. **(B)** Footprinting of PNI model rats in the CC, MPC, and MPC@BDNF groups. **(C)** Representative three-dimensional foot pressure maps of the PNI models following repair among the groups. **(D)** SFI statistical plot (ns: no significant difference, *p < 0.05, **p < 0.01).

Furthermore, to conduct a more comprehensive assessment of the impact of quantifying the MPC on the facilitation of nerve regeneration in the sciatic nerve and subsequent motor function recovery, the gait trajectories and plantar pressure stress maps of the experimental rats were examined at 12 weeks post-surgery. This analysis was performed utilizing the CatWalk gait analyzer (Figure 9A). The representative images of footprints and 3D plantar pressure of the MPC, MPC@BDNF, and CC groups revealed that the footprints and plantar pressure patterns exhibited similar trends in the rats belonging to the MPC and MPC@BDNF groups. Moreover, the toe extension, plantar pressure, and contact area of both the MPC and MPC@BDNF groups were greater than those of the CC group (Figures 9B,C).

The SFI was utilized as an impartial metric for assessing the process of restoring motor ability in rats. Throughout the course of the experiment, the researchers documented the footprints left by the rats as they traversed the pathway. Subsequently, the SFI was computed for each rat using the appropriate formula. The results of the study revealed that both the group receiving MPC@BDNF and the group receiving MPC showed a notable increase in the SFI in comparison to the CC. However, there was no significant difference in the SFI between the MPC@BDNF group and the MPC group. Furthermore, the SFIs of MPC@BDNF and CC were significantly different, as were the SFIs of MPC and CC (Figure 9D).

The findings from our histological study and behavioral tests indicate that both MPC and MPC@BDNF demonstrate significant efficacy in enhancing the functional restoration of peripheral nerves in SD rats with 15 mm sciatic nerve injury.

## 4 Discussion

Functional rehabilitation from PNI is a key problem in regenerative medicine (Vijayavenkataraman, 2020) because nerve grafting has limitations and risks (Yi et al., 2019; Fadia et al., 2020; Modrak et al., 2020). The nerve conduit fills the gap of the traditional method. Researchers have devised several methodologies to enhance the effectiveness of neural regeneration, hence advancing the field of nerve conduits, such as combining growth factors, drugs, and cells (Wang et al., 2020), to enhance axon regeneration and myelin regeneration (Mackinnon and Hudson, 1992; Cattin et al., 2015; Huang et al., 2021; Yu et al., 2021). However, these strategies do not provide an optimal environment or biomechanical support for nerve regeneration and need further improvement (Carriel et al., 2013; Du et al., 2017; Sun et al., 2018). Therefore, the investigation of a nerve conduit technique that facilitates both nerve regeneration and self-docking is highly important.

In nature, mussels firmly adhere to the surfaces of reefs, ships, and other objects, despite the constant strong waves at the seaside, inspired by the superior adhesion mechanisms derived from marine mussels (Waite, 1983; Waite and Qin, 2001; Choi et al., 2011). The mussel mimetic polymer polydopamine, which closely resembles that of native tissues, has attracted great attention for a diverse array of applications within the field of biomedical engineering (Waite, 2002; Lee et al., 2007; Ku et al., 2010). Therefore, in our study, the selection of a mussel mimetic polymer with excellent and unique adhesion properties could reduce the traditional operation time and number of suture injuries, thereby reducing the occurrence of neuroma and infection and creating better conditions suitable for peripheral nerve tissue regeneration (Lee et al., 2006; Mehdizadeh et al., 2012; Annabi et al., 2014; Azuma et al., 2015; Kim et al., 2017), thus compensating for the lack of properties of traditional synthetic polymers (Cha, Hwang, and Lim, 2008; Daly et al., 2013; Panagopoulos, Megaloikonomos, and Mavrogenis, 2017).

Most biodegradable and biocompatible nerve conduits have hollow structures (Zhang et al., 2019). To further improve the regenerative effect and efficiency of injured nerves, appropriate nerve growth factors have been designed to fill the lumen to construct a beneficial luminal microenvironment (Gao et al., 2016). BDNF and its associated receptors exhibit extensive expression across the nervous system, facilitating neuronal growth and synaptic formation in the brain (Allen et al., 2013). Consequently, they have found broad application in the management of cerebral ischemia‒reperfusion injury and peripheral nerve regeneration (Han et al., 2011; Autry and Monteggia, 2012; Wang, Yuan, et al., 2016). BDNF plays a crucial role in the maintenance of myelination and the regeneration of axons subsequent to PNI. This mechanism has the potential to greatly enhance the velocity and efficacy of nerve regeneration, hence facilitating the recovery process following injury (Zuccato and Cattaneo, 2009; Nagahara and Tuszynski, 2011). The exploration of its function in the body’s nervous system and its role at both the molecular and cellular levels has been the focus of extensive research.

Consequently, we investigated novel therapeutic interventions and biomaterials aimed at enhancing the regenerative potential of PNI. Replicating the neural milieu inside a nerve conduit with synergistically applied neurotrophins is crucial for promoting peripheral nerve regeneration. To address this requirement, we successfully designed nerve catheters loaded with BDNF. Notably, our approach does not necessitate the use of particular instruments, complex procedures, or stringent conditions for the reaction. To evaluate the effectiveness of sciatic nerve injury research in a rat model, it is important to perform an assessment. The multitude of advantages and distinctive qualities associated with it have led to its heightened use across diverse fields, including biological and biomaterial sciences. The adhesive properties of MPC@BDNF can replace traditional suture techniques so that nerve endings can be effectively connected without complicated suture procedures, which can provide physical support for peripheral nerve regeneration (Cheong et al., 2019). This methodology decreases the need for complex technical expertise and enables expedited surgical procedures with little tissue damage and inflammatory reactions, consequently enhancing the efficacy of nerve restoration. The present study demonstrated a notable improvement in the nerve microenvironment in the MPC@BDNF group compared with the CC and MPC groups. This improvement has positive implications for axon regeneration, muscle fiber remodeling, and the recovery of motor function in innervated muscles. Furthermore, the MPC@BDNF had a synergistic effect on peripheral nerve regeneration, as depicted in Figures 5, 6. Its good antibacterial properties also reduced the occurrence of surgical infection. The possible serious consequences were avoided. To the best of our knowledge, we are the first to develop and apply the MPC@BDNF nerve conduit for regeneration of peripheral nerves.

## 5 Conclusion

In summary, we were the first to successfully prepare MPC@BDNF, which was explored in the repair and regeneration of a 15 mm injury model of PNI. MPC@BDNF has good material characteristics, and its excellent mechanical properties and adhesion properties can reduce the duration of routine surgery and reduce the occurrence of postoperative complications so that patients can benefit the most. Our *in vitro* tests showed that the MPC@BDNF group was comparable to the other groups of cells. Moreover, previous *in vivo* investigations have demonstrated that the introduction of MPC@BDNF into rats over a period of 12 weeks can effectively promote the development and progression of differentiating nerve cells. This intervention also facilitates the regeneration of axons and the myelin sheath, ultimately resulting in successful restoration of the sciatic nerve and motor ability. Furthermore, the observed outcomes indicate favorable histocompatibility of the implanted material. Hence, this study presents a potentially effective nerve conduit for the regeneration of peripheral nerves subsequent to injury. This study highlights the significant practical implications of utilizing MPC@BDNF for nerve regeneration and addresses the existing research gap in its application within the PNI model. The assessment of the cytocompatibility and histology of MPC@BDNF is crucial for advancing our understanding of its mechanism. This knowledge will contribute significantly to the evolution of personalized nerve conduits in the domain of neural engineering in combination with the discipline of regenerative medicine. Furthermore, these findings hold great potential for clinical translation and application.

## Data Availability

The original contributions presented in the study are included in the article/Supplementary Material, further inquiries can be directed to the corresponding author.
