# Supplementary figures and images for: BDNF-loaded chitosan-based mimetic mussel polymer conduits for repair of peripheral nerve injury
Source: Front Cell Dev Biol. 2024 Jul 1;12:1431558. doi: 10.3389/fcell.2024.1431558 (PMC11246889; doi:10.3389/fcell.2024.1431558)

Figure 1


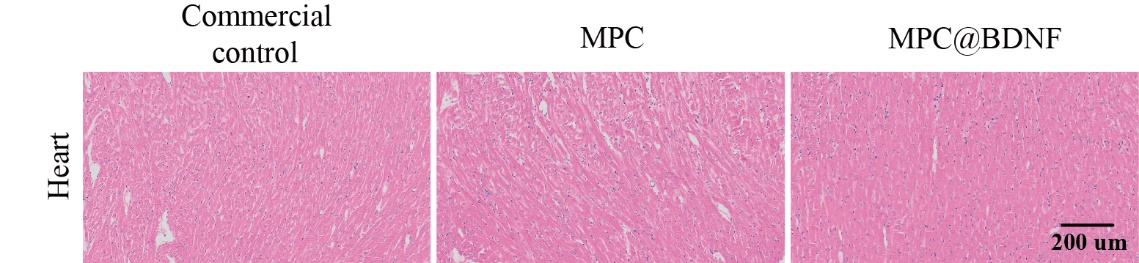


Figure 2


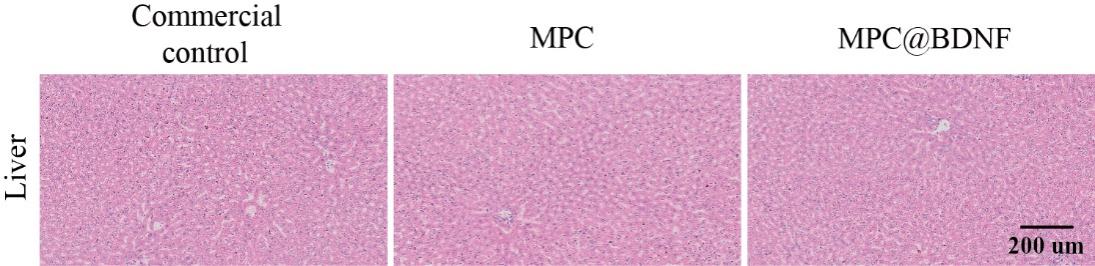


Figure 3


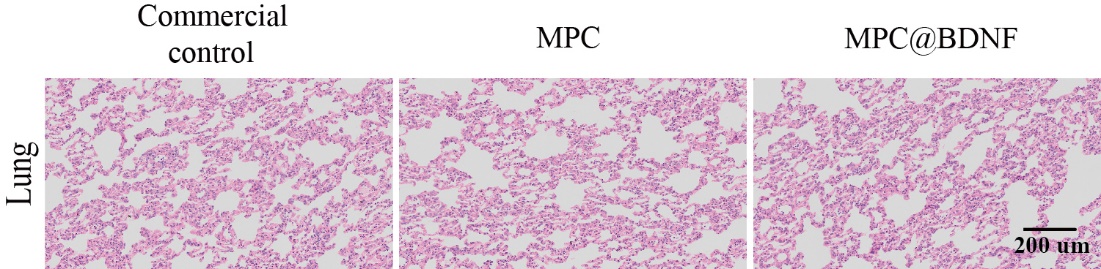


Figure 4


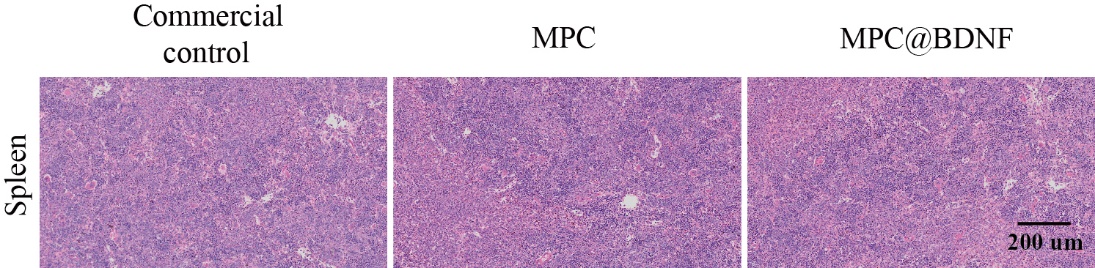


Figure 5


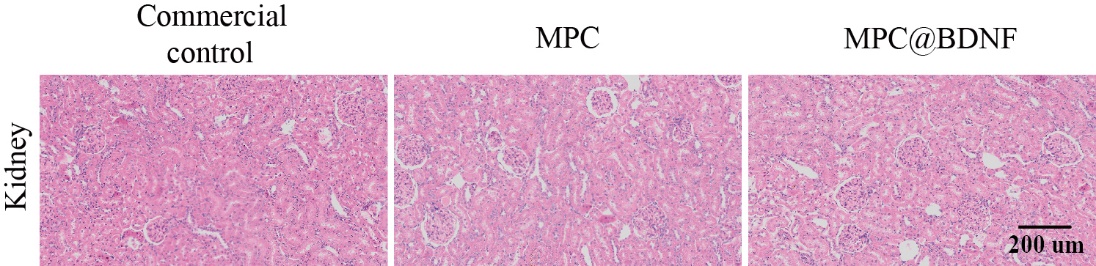

Supplement: Supplementary file 1 [file Table1.DOCX]
